# Transcriptome analysis of a taxol-producing endophytic fungus *Cladosporium cladosporioides* MD2

**DOI:** 10.1186/s13568-018-0567-6

**Published:** 2018-03-19

**Authors:** Li-Yun Miao, Xin-Chun Mo, Xiao-Yuan Xi, Lan Zhou, Ge De, You-Sheng Ke, Pan Liu, Fa-Jun Song, Wen-Wen Jin, Peng Zhang

**Affiliations:** 1 Hubei Provincial Key Laboratory for Protection and Application of Special Plants in Wuling Area of China, Key Laboratory of State Ethnic Affairs Commission for Biological Technology, College of Life Science, South-Central University for Nationalities, Wuhan, 430074 China; 2 School of Life Science, Wuchang University of Technology, Wuhan, 430074 China; 3 School of Life Science and Technology, Huazhong University of Science and Technology, Wuhan, 430074 China; 4 School of Applied Technology, Lijiang Teacher College, Lijiang, 674100 China

**Keywords:** Endophytic fungus, *Cladosporium cladosporioides*, Transcriptome, Taxol

## Abstract

The shortage of molecular information for taxol-producing fungi has greatly impeded the understanding of fungal taxol biosynthesis mechanism. In this study, the transcriptome of one taxol-producing endophytic fungus *Cladosporium cladosporioides* MD2 was sequenced for the first time. About 1.77 Gbp clean reads were generated and further assembled into 16,603 unigenes with an average length of 1110 bp. All of the unigenes were annotated against seven public databases to present the transcriptome characteristics of *C. cladosporioides* MD2. A total of 12,479 unigenes could be annotated with at least one database, and 1593 unigenes could be annotated in all queried databases. In total, 8425 and 3350 unigenes were categorized into 57 GO functional groups and 262 KEGG pathways, respectively, exhibiting the dominant GO terms and metabolic pathways in the *C. cladosporioides* MD2 transcriptome. One potential and partial taxol biosynthetic pathway was speculated including 9 unigenes related to terpenoid backbone biosynthesis and 40 unigenes involved in the biosynthetic steps from geranylgeranyl diphosphate to 10-deacetylbaccatin III. These results provided valuable information for the molecular mechanism research of taxol biosynthesis in *C. cladosporioides* MD2.

**Electronic supplementary material:**

The online version of this article (10.1186/s13568-018-0567-6) contains supplementary material, which is available to authorized users.

## Introduction

Taxol (also named as Paclitaxel) originally isolated from *Taxus brevifolia* (Wani et al. 1971) was well-known as an anti-cancer drug used in clinic. Fermentation processes using taxol-producing microorganisms were considered as an efficient and sustainable way to produce taxol (Gond et al. 2014; Choi et al. 2016). Since one taxol-producing endophytic fungus *Taxomyce andreanae* isolated from the bark of *T. brevifolia* was firstly reported (Stierle et al. 1993), more than 40 fungal genera about 200 endophytic fungi had been reported to produce taxol (Flores-Bustamante et al. 2010; Kusari et al. 2014). However, low and un-stable taxol productivity in fungi restricted their industrial application (Gond et al. 2014; Kusari et al. 2014). Targeted genetic engineering of taxol-producing fungi was an effective way to exploit their taxol productivity, however there were difficult challenges due to the almost complete lack of molecular information on the exact pathway, key rate-limiting steps, and availability of a complete set of genes involved in the taxol biosynthesis of fungi (Flores-Bustamante et al. 2010; Gond et al. 2014). Thus, a prerequisite was to discover taxol-related genes and uncover the molecular mechanism of taxol biosynthesis in fungi (Flores-Bustamante et al. 2010; Kusari et al. 2014).

High-throughput RNA-seq technology has been extensively used for transcriptome analysis of many organisms without reference genomes to discover novel and interesting genes (Grabherr et al. 2011; Zhang et al. 2013). Some of representative examples were that several transcriptomes of *Taxus* were investigated with RNA-seq technology to comprehensively reveal candidate genes involved in taxol biosynthesis. da Hao et al. (2011) used RNA-seq technology to sequence the leaf transcriptome of *Taxus mairei*, and obtained 36,493 unigenes, including hundreds of taxane biosynthetic genes and some genes involved in tissue specific functions. Li et al. (2012) used RNA-seq technology to investigate the transcriptional profile of *Taxus chinensis* cells in response to methyl jasmonate (MeJA) elicitation, obtained 46,581 unigenes, and detected 13,469 differentially expressed genes, including all of the known jasmonate (JA) biosynthesis/JA signaling pathway genes and taxol-related genes. Sun et al. (2013) used RNA-seq technology to investigate the transcriptomes of MeJA-treated and un-treated *Taxus x media* cells, obtained 40,348 unigenes, detected all the 29 known genes involved in the biosynthesis of terpenoid backbone and taxol, and found multiple candidates for the unknown steps in taxol biosynthesis. Additionally, Qiao et al. (2014) used RNA-seq technology to sequence the leaf transcriptome of *Cephalotaxus hainanensis*, obtained 39,416 unigenes, and detected a number of putative candidate genes involved in taxol biosynthesis. More importantly, most of taxol-related genes in *Taxus* have been cloned and characterized in vitro by functional expression in *Escherichia coli* or yeast using ‘surrogate’ substrates, and one taxol biosynthesis pathway in *Taxus* consisting of about 20 biosynthetic enzymes was depicted (Jennewein and Croteau 2001; Walker and Croteau 2001; Croteau et al. 2006; Guo et al. 2006; Liu et al. 2016). These fruitful results gave us a relatively comprehensive knowledge of the taxol biosynthesis in *Taxus.* However, little was known about the fungal taxol biosynthesis pathway and taxol-related genes in fungi.

Recently, Yang et al. (2014) sequenced the genome of one taxol-producing fungus *Penicillium aurantiogriseum* NRRL 62431 isolated from *Corylus avellana* L., identified candidate genes involved in taxol biosynthesis, and found low sequence identities between the taxol biosynthetic candidate genes of *P. aurantiogriseum* NRRL 62431 and that of its host hazel as well as that of *Taxus baccata*. However, the molecular information on the taxol biosynthesis of *Cladosporium cladosporioides* MD2, one taxol-producing fungus isolated from *T. x media* (Zhang et al. 2009), was almost completely lacking. In this study, the transcriptome of *C. cladosporioides* MD2 was firstly sequenced by Illumina RNA-seq technology to generate 19,714,114 clean reads, that were de novo assembled with Trinity program (Grabherr et al. 2011) into 16,603 unigenes. All of the unigenes were annotated and analyzed by using BLAST against various protein and nucleotide databases, and the biological functions and metabolic pathways of these unigenes were further characterized through Gene Ontology (GO) annotation and Kyoto Encyclopedia of Genes and Genomes (KEGG) annotation. One potential and partial taxol biosynthesis pathway in *C. cladosporioides* MD2 was speculated, and dozens of unigenes were annotated to be involved in taxol biosynthesis. These results provided a valuable source for further gene discovery, functional genomic research and comparative transcriptome analysis of *C. cladosporioides* MD2.

## Materials and methods

### Fungal material and RNA extraction

*Cladosporium cladosporioides* MD2 was identified by China Center for Type Culture Collection (CCTCC) and deposited under CCTCC no. 207063 (Zhang et al. 2009). One milliliter of the spore suspension (about 10^8^ spores) of *C. cladosporioides* MD2 was cultivated in 100 mL of potato dextrose liquid medium at 28 °C and 150 rpm for 132 h (equal to five and a half days), and the mycelium pellets were collected by centrifugation for RNA extraction. According to the manufacturer’s instruction, total RNA was extracted by using TRIzol^®^ Reagent (Invitrogen, California, USA), treated with DNase I (Invitrogen, California, USA), dissolved in RNase-free water, and stored in a − 80 °C freezer. The quality and integrity of total RNA were detected by using Nanodrop2000 spectrophotometer (Thermo Fisher Scientific, California, USA) and Bioanalyzer 2100 (Agilent, California, USA), respectively. The purity of total RNA was assessed by both A260/280 and A260/230 ratios.

### cDNA synthesis and library sequencing

About 60 μg of total RNA at a concentration ≥ 600 ng/μL, OD260/280 = 1.8 ~ 2.2 and RNA integrity number (RIN) ≥ 7.0 were used for construction of a cDNA sequencing library using a TruSeqTM RNA Sample Preparation Kit (Illumina, San Diego, California, USA). Briefly, poly-A mRNA samples were isolated from the total RNA using oligo (dT) attached magnetic beads and then interrupted into 200–500 bp fragments. Subsequently, the fragmented mRNAs as well as random hexamer primers and reverse transcriptase were used to synthesize first-strand cDNAs, that were used to synthesize second-strand cDNAs with DNA polymerase I and RNase H. The cDNA products were purified with QIAquick PCR Purification Kit (Qiagen, California, USA), end-repaired and A-tailed using End Repair Mix (Qiagen, California, USA), and then ligated with sequencing index adapters (Illumina, San Diego, California, USA). The ligated products were isolated by gel electrophoresis to select suitable fragments and were subsequently amplified to generate the final cDNA libraries, that were sequenced using Illumina HiSeq™ 2500 sequencing platform at Wuhan Bio-Broad Biotechnology Co., Ltd. (Wuhan, China).

### De novo assembly by trinity

The raw reads were filtered to generate clean reads by removal of adaptors, ambiguous reads with unknown nucleotides larger than 10%, and low-quality reads where more than half of the bases’ qualities were less than five (Quality score < 5). The clean reads were deposited in NCBI Sequence Read Archive (SRA) Sequence Database with accession number SRR6147052. Subsequently, the clean reads were de novo assembled by Trinity program (Grabherr et al. 2011) to generate non-redundant unigenes. In brief, all the clean reads were assembled into contigs with different k-mer values to assess optimal assembly range. The assembled transcripts were as reference sequences for transcript annotation and re-validated by aligning clean reads back to transcripts (Miller et al. 2012). Each of clean reads was re-mapped to reference sequences using Bowtie (Langmead 2010) with default parameters. The longest transcripts within each operationally assembled contig were defined as unigenes.

### Transcriptome annotation

The annotation of unigenes were performed by using BLAST algorithm against NCBI non-redundant protein sequence (Nr) database, NCBI non-redundant nucleotide sequence (Nt) database, Swiss-Prot database, Protein family (Pfam) database with an E-value threshold of 1.0E−5, and the best aligning results were used to annotate unigenes and decide the sequence direction of each unigene. Additionally, some unigenes that had no alignment hit in the above databases were further estimated by using ESTScan (3.0.3) software (Iseli et al. 1999) to predict their protein coding sequences (CDS). With Nr and Swiss-Prot annotation, Blast2GO software (Conesa et al. 2005) was used to perform gene ontology (GO) annotation of unigenes to obtain cellular component, molecular function and biological process terms. After retrieving the associated GO terms, the possible functions of unigenes were predicted against eukaryotic Orthologous Group (KOG) database. Additionally, the Kyoto Encyclopedia of Genes and Genomes (KEGG) pathways annotation of unigenes was performed by mapping the sequences obtained from Blast2GO to the contents of KEGG database (Kanehisa and Goto 2000).

### Analysis of potential fungal taxol biosynthesis pathway

The peptide sequences of unigenes were searched on Carbohydrate-Active Enzymes (CAZy) database (Park et al. 2010) to find the enzymes involved in taxol biosynthesis pathway. In parallel, the reported gene sequences involved in the taxol biosynthesis from *Taxus* or other organisms were retrieved from GenBank. All the sequences were then manually aligned by BLAST search against the assembled contigs to predict potential genes involved in the taxol biosynthesis. All the hits with an E-value threshold of 1.0E−10 were used as queries to search KEGG database again for further identification, and the genes were kept if their subjects were annotated as the enzymes involved in the taxol biosynthesis.

### Reverse-transcribed PCR validation of unigenes

Total RNA from *C. cladosporioides* MD2 was reverse-transcribed by using SuperScript III Reverse Transcriptase (Invitrogen, California, USA) and oligo(dT)18. Ten of assembled unigenes were randomly selected for reverse-transcribed PCR (RT-PCR) validation, and the primer sequences of these assembled unigenes were listed in Additional file 1: Table S1.

## Results

### Transcriptome sequencing and assembly

**Fig. 1:**
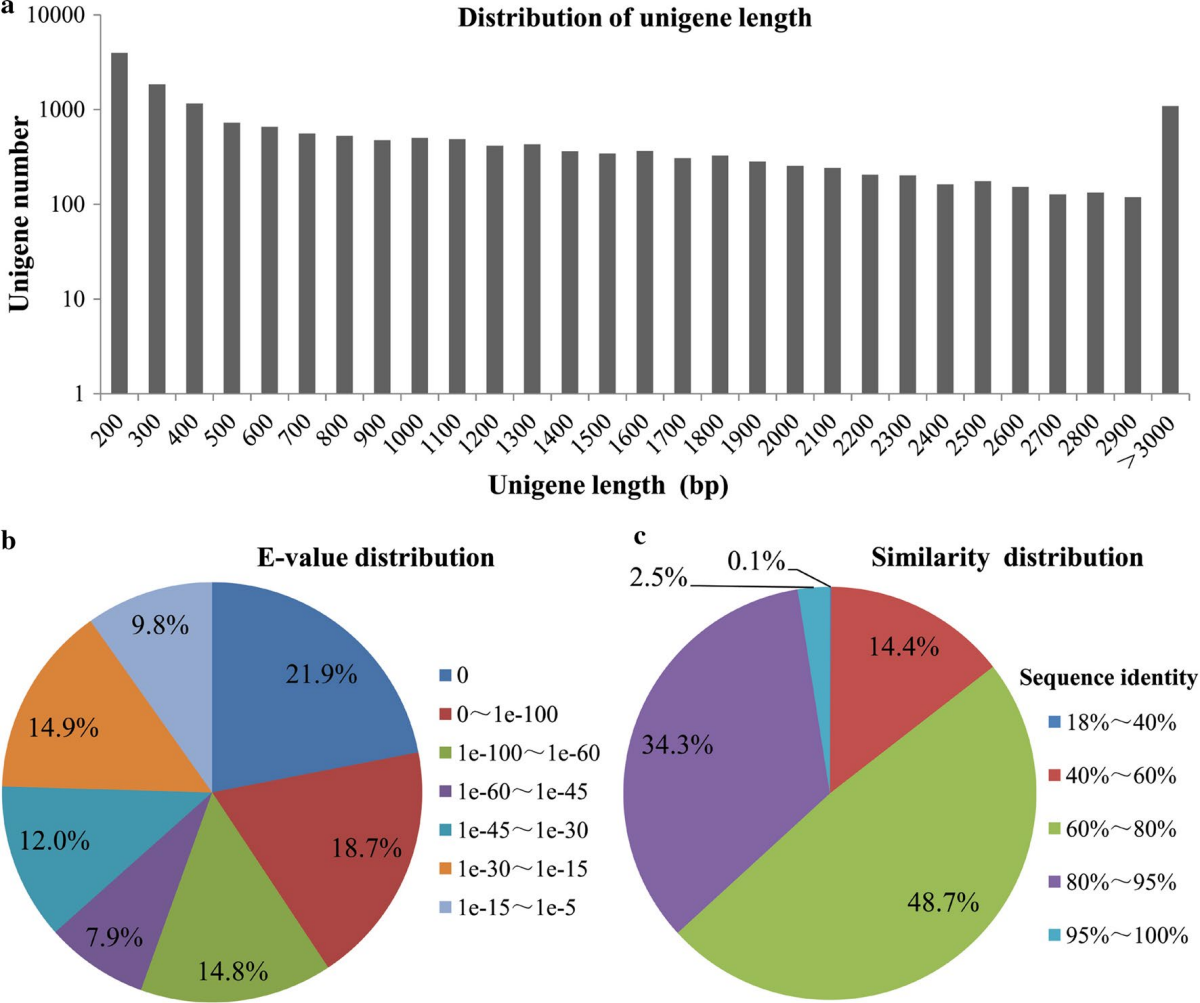
mRNA-seq and de novo assembly of *C. cladosporioides* MD2 transcriptome. **a** Distribution of unigene length. **b** E-value distribution of BLAST hits for each unique sequence against the Nr database. **c** Similarity distribution of the top BLAST hits for each sequence against the Nr database

The transcriptome of *C. cladosporioides* MD2 was sequenced with Illumina sequencing technology to generate 19,776,638 raw reads consisting of 1,779,897,420 nucleotides (nt). After removing the adaptor sequences, ambiguous reads and low-quality reads, a total of 19,714,114 clean reads (approximate 1.77 Gbp) were obtained (GenBank Accession Number: SRR6147052), and were further de novo assembled into 16,603 unigenes with an average length of 1110 bp and an N_50_ of 1894 bp (Table 1). Among them, 6976 unigenes, 2942 unigenes and 3819 unigenes were within the ranges of 200–500, 500–1000 and 1000–2000 bp, respectively, and 2866 unigenes were longer than or equal to 2000 bp (Fig. 1a). Additionally, ten of the assembled unigenes of *C. cladosporioides* MD2 were randomly selected for RT-PCR validation, and all of the 10 assembled unigenes got right amplifications (Additional file 1: Figure S1). These results indicated the suitability of mRNA-seq for extensive and accurate assembly of the *C. cladosporioides* MD2 transcriptome.

**Table 1:** Assembly summary of *C. cladosporioides* MD2 unigenes

Assembly	Number
Number of raw reads	19,776,638
Number of clean reads	19,714,114
Total unigenes	16,603
N_50_ length (bp)	1894
Mean length (bp)	1110
Min length (bp)	201
Max length (bp)	10,055
Unigenes with 200 ~ 500 bp	6976
Unigenes with 500 ~ 1000 bp	2942
Unigenes with 1000 ~ 2000 bp	3819
Unigenes with ≥ 2000 bp	2866

### Transcriptome annotation

All of the 16,603 unigenes were annotated by BLAST alignment against different protein and nucleotide databases. A total of 11,670 unigenes (70.29%) had homologous sequences in the Nr database (Table 2), suggesting that most of the unigenes could be translated into proteins. Based on the annotation results of Nr database, E-value distribution analysis showed that 75.30 and 63.30% of the matched sequences had strong homology with E-value < 1.0E−30 and E-value < 1.0E−45, respectively, and whereas only 24.5% of the matched sequences had high similarity with E-value from 1.0E−30 to 1.0E−5 (Fig. 1b). Similarity distribution analysis showed that 36.80% of the mapped sequences had a similarity higher than 80%, whereas 63.2% of the mapped sequences had a similarity ranging from 18 to 80% (Fig. 1c). Additionally, species distribution analysis revealed that *C. cladosporioides* MD2 had a number of homologous sequences with several species, and the genes from *Baudoinia compniacensis* had the highest similarity (19.90%) with that from *C. cladosporioides* MD2, followed by *Dothistroma septosporum* (14.50%), *Pseudocercospora fijiensis* (10.90%), *Zymoseptoria brevis* (8.40%), and *Zymoseptoria tritici* (7.10%). The low sequence similarity of the genes from *C. cladosporioides* MD2 to the genes from these above species suggested the impossibility of using the genomes of these relative species as references for the transcriptome analysis of *C. cladosporioides* MD2.

**Table 2:** Summary of *C. cladosporioides* MD2 unigene annotations

Databases	Number of mapped unigenes	Mapping rates (%)
Nr	11,670	70.29
Nt	3048	18.35
KEGG	3350	20.17
SwissProt	7211	43.43
Pfam	8431	50.77
GO	8425	50.74
KOG	4904	29.53
Annotated by all seven databases	1593	9.59
Annotated by at least one database	12,479	75.16

Additionally, 3048 unigenes (18.35%), 8431 unigenes (50.78%), 7211 unigenes (43.43%), 8425 unigenes (50.74%), 4904 unigenes (29.53%) and 3350 unigenes (20.17%) were annotated in the databases of Nt, Pfam, Swiss-Prot, GO, KOG and KEGG, respectively, and the mapping rates were from 18 to 71% (Table 2). In total, 12,479 unigenes (75.16%) could be annotated with at least one database, and 1593 unigenes (9.59%) could be annotated in all of seven queried databases, with relatively well defined functional annotations (Table 2). These annotations gave us a valuable resource for investigating specific pathways and function genes in *C. cladosporioides* MD2. Furthermore, all of the unigenes were aligned by BLASTX (E-value ≤ 1.0E−5) to all of seven queried databases to predict their coding sequences (CDS), and the correct reading frames of the nucleotide sequences of unigenes (5′-3′ direction) were defined by the highest rank in the BLAST results. In addition, the CDS of some unigenes, that could not be aligned to the above databases, were predicted by ESTScan software. In total, 16,578 unigenes were identified to harbor their CDS, where the CDS of 11,975 unigenes were identified by BLAST alignment and that of 4603 were estimated by the ESTScan.

### GO and KOG classification of unigenes

**Fig. 2:**
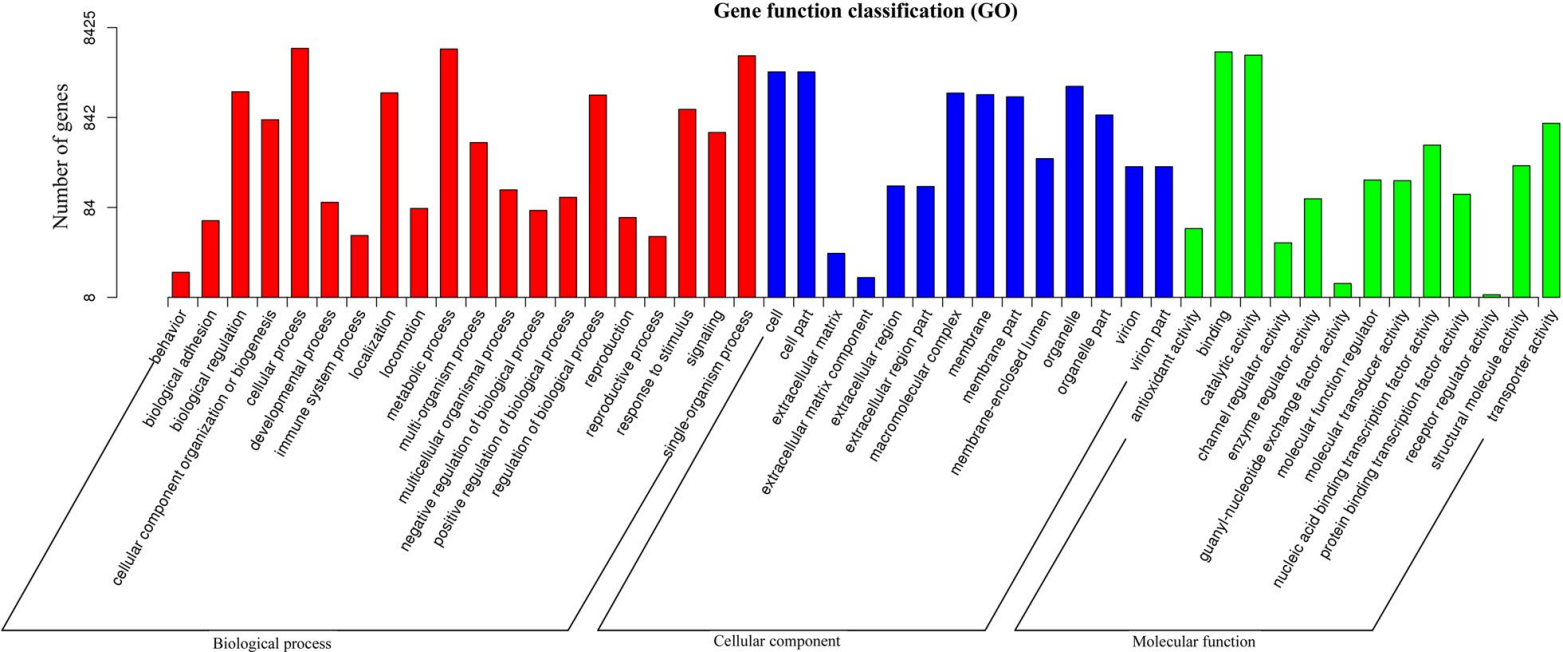
GO annotation of the *C. cladosporioides* MD2 unigenes. The annotated unigenes were classified into cellular component, molecular function and biological process categories

**Fig. 3:**
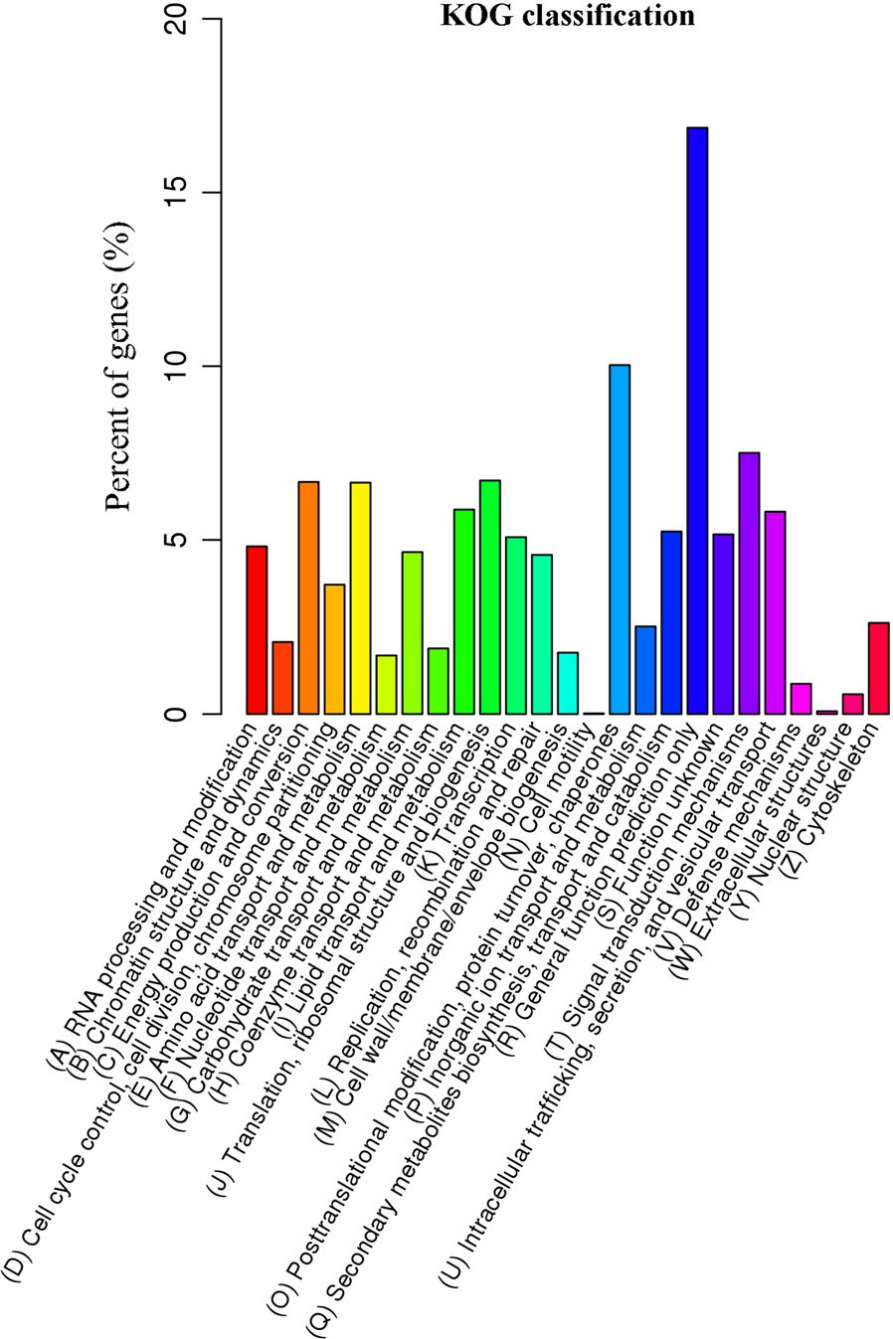
KOG classification of the *C. cladosporioides* MD2 unigenes. The left y-axis indicated the percent of genes in a category. The dominating categories included ‘general function predictions only’ (827 unigenes), ‘post-translational modification, protein turnover, chaperones’ (492 unigenes), ‘signal transduction mechanisms’ (368 unigenes), ‘translation, ribosomal structure and biogenesis’ (329 unigenes), and ‘energy production and conversion’ (327 unigenes)

To classify the predicted function of all unigenes, Blast2GO software was employed to search against GO database and KOG database. A total of 8425 unigenes were categorized into 57 functional groups under three GO main categories, including ‘Biological Process’, ‘Cellular Component’ and ‘Molecular Function’. The ‘Biological Process’ category occupied the largest proportion (25 terms), followed by ‘Cellular Component’ (18 terms) category and ‘Molecular Function’ category (14 terms) (Fig. 2; Additional file 1: Table S2). In the ‘Biological Process’ category, the major GO terms included ‘cellular process’ (4944 unigenes), ‘metabolic process’ (4861 unigenes), ‘single-organism process’ (4090 unigenes), ‘biological regulation’ (1627 unigenes), ‘localization’ (1583 unigenes) and ‘regulation of biological process’ (1496 unigenes). In the ‘Cellular Component’ category, the representative terms included ‘cell’ (2707 unigenes), ‘cell part’ (2707 unigenes), ‘organelle’ (1870 unigenes), ‘macromolecular complex’ (1575 unigenes), ‘membrane’ (1512 unigenes) and ‘membrane-part’ (1429 unigenes). In the ‘Molecular Function’ category, the dominant GO terms were ‘binding’ (4519 unigenes), ‘catalytic activity’ (4156 unigenes), ‘transporter activity’ (726 unigenes) and ‘nucleic acid binding transcription factor activity’ (415 unigenes). A high percentage of genes (more than 4000 unigenes) were categorized into ‘cellular process’, ‘metabolic process’, ‘single-organism process’, ‘binding’ and “catalytic activity”, demonstrating the dominated biological activities being taking place in the *C. cladosporioides* MD2 transcriptome. In addition, 4904 unigenes were categorized (E-value ≤ 1.0E−5) in 25 functional KOG clusters (Fig. 3), and the five largest categories were ‘general function predictions only’ (827 unigenes), ‘post-translational modification, protein turnover, chaperones’ (492 unigenes), ‘signal transduction mechanisms’ (368 unigenes), ‘translation, ribosomal structure and biogenesis’ (329 unigenes) and ‘energy production and conversion’ (327 unigenes). These results demonstrated the major biological functions of the genes expressed in the *C. cladosporioides* MD2 transcriptome.

### KEGG analysis of unigenes

**Fig. 4:**
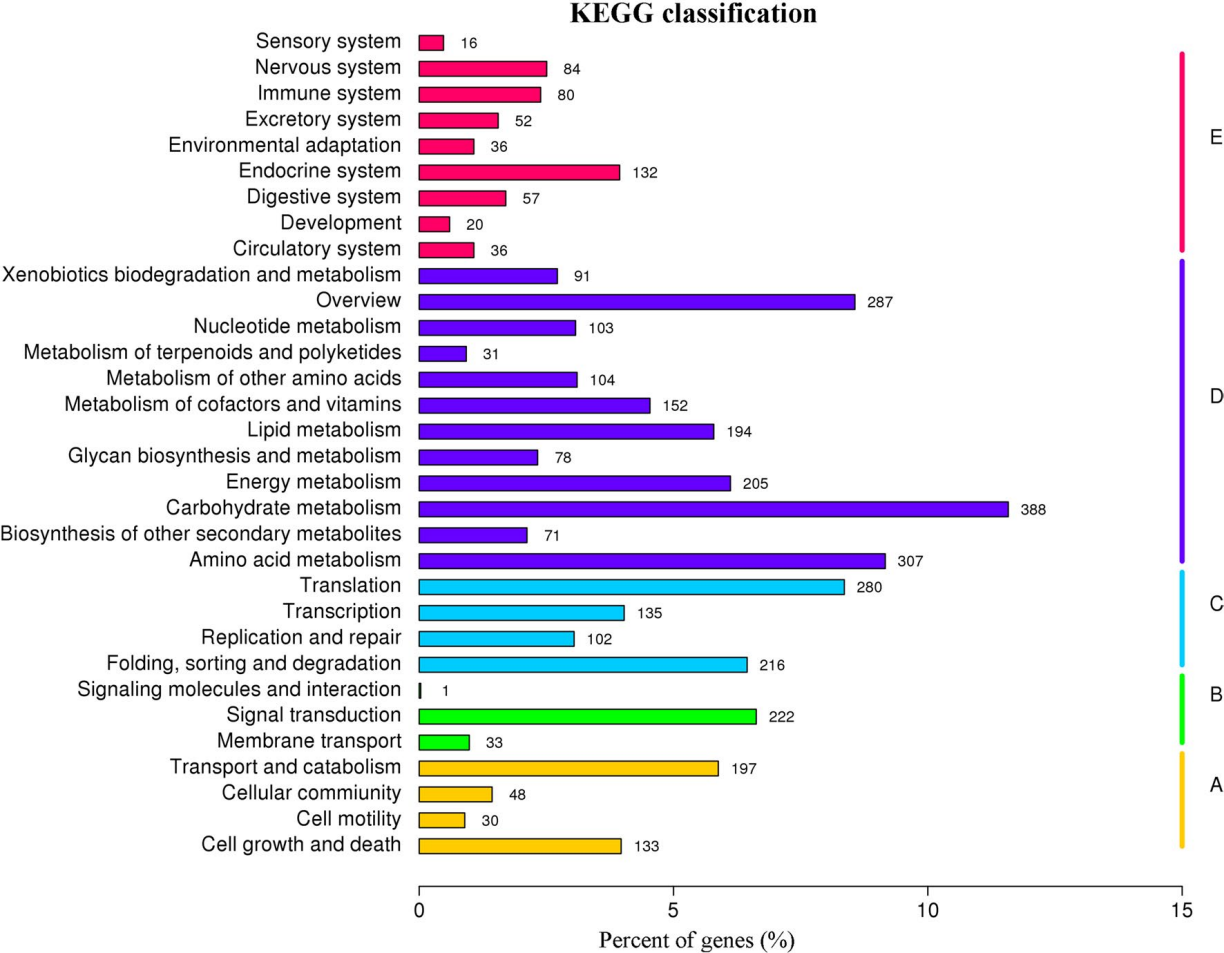
KEGG classification of the *C. cladosporioides* MD2 unigenes. **a** Cellular processes; **b** environmental information processing; **c** genetic information processing; **d** metabolism; **e** organismal systems. The numbers on the column represented the estimation of unigenes within the pathway

To better understand the active genes involved in the biological pathways of *C. cladosporioides* MD2, all of the unigenes were mapped to KEGG database. A total of 3350 unigenes were assigned to 262 KEGG pathways, and the top five pathways hierarchy (ranked by unigene numbers) were ‘Carbohydrate metabolism’ (388 unigenes), ‘Amino acid metabolism’ (307 unigenes), ‘Overview’ (287 unigenes), ‘Translation’ (280 unigenes) and ‘Signal transduction’ (222 unigenes) (Fig. 4). The dominant KEGG pathways included ‘biosynthesis of amino acids’ (157 unigenes, ko01230), followed in sequence by ‘carbon metabolism’ (150 unigenes, ko01200), ‘ribosome’ (104 unigenes, ko03010), ‘spliceosome’ (97 unigenes, ko03040), ‘purine metabolism’ (79 unigenes, ko00230), ‘cell cycle-yeast’ (78 unigenes, ko04111) and ‘starch and sucrose metabolism’ (78 unigenes, ko00500) (Additional file 1: Table S3), suggesting that most of the genes expressed in the *C. cladosporioides* MD2 transcriptome were involved in basic metabolic pathways.

**Fig. 5:**
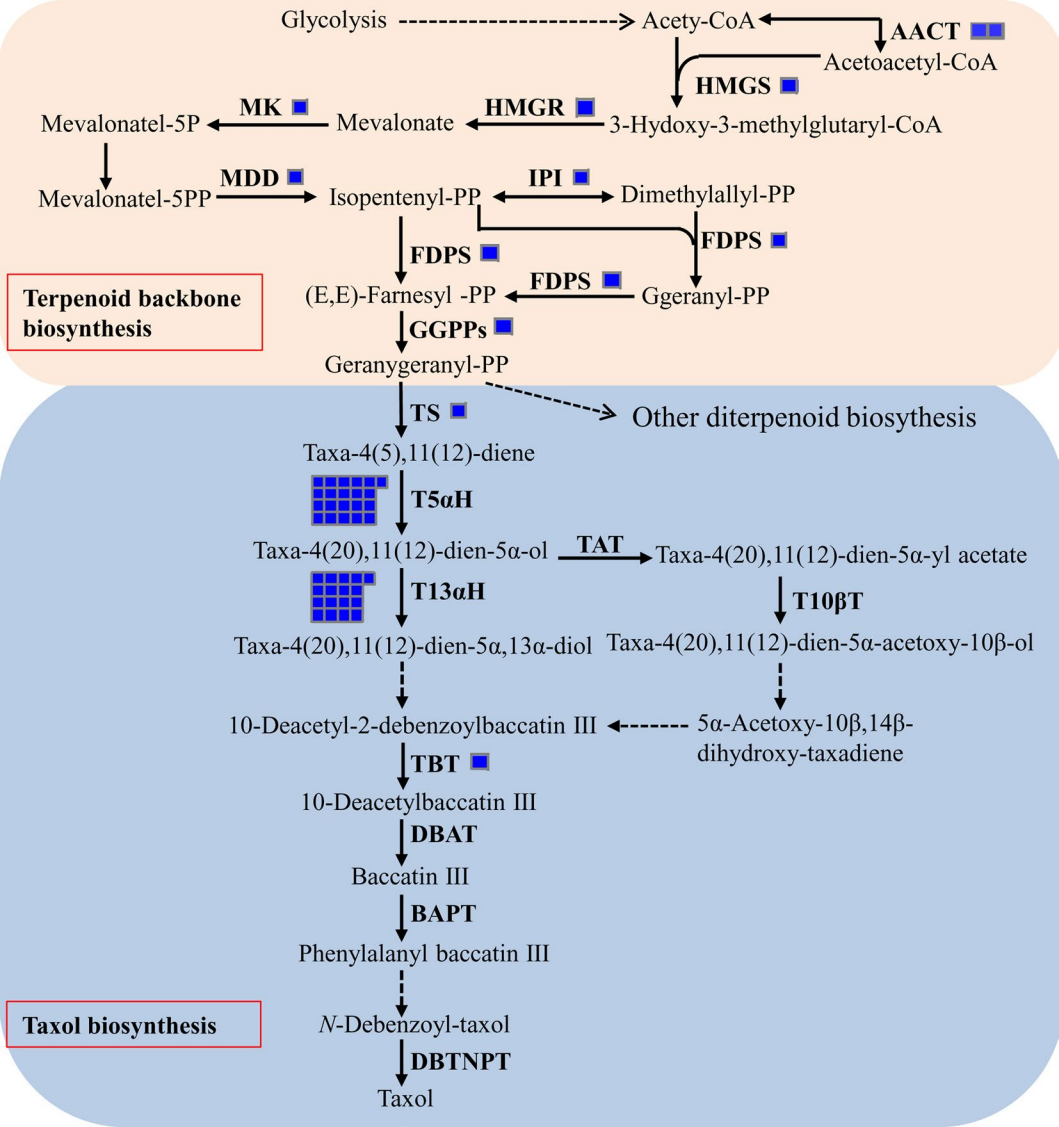
Overview of the potential fungal taxol biosynthesis pathway of *C. cladosporioides* MD2. The numbers of genes in families with at least one gene were indicated by the numbers of blue rectangles. Identified enzymes included: *AACT* acetyl-CoA C-acetyltransferase, *HMGS* hydroxymethylglutaryl-CoA synthase, *HMGR* hydroxymethylglutaryl-CoA reductase, *MK* mevalonate kinase, *MDD* diphosphomevalonate decarboxylase, *IPI* isopentenyl-diphosphate delta-isomerase, *FDPS* farnesyl diphosphate synthaseisopentenyl-diphosphate delta-isomerase, *GGPPs* geranylgeranyl diphosphate synthaseisopentenyl-diphosphate delta-isomerase, *TS* taxadiene synthaseisopentenyl-diphosphate delta-isomerase, *T5αH* taxadiene 5-alpha-hydroxylaseisopentenyl-diphosphate delta-isomerase, *T13αH* taxane 13-alpha-hydroxylaseisopentenyl-diphosphate delta-isomerase, *TBT* 2-alpha-hydroxytaxane 2-*O*-benzoyltransferase

Dozens of unigenes were detected to be involved in ‘diterpenoid biosynthesis’. Among them, 9 unigenes were involved in terpenoid backbone biosynthesis (Fig. 5; Additional file 1: Table S4). Unigenes of c12899_g1 and c8934_g1 were homologous to an acetyl-CoA C-acetyltransferase (AACT) gene. Unigenes of c13511_g1, c14615_g1, c12988_g1 and c10355_g1 were homologous to the genes for hydroxymethylglutaryl-CoA synthase (HMGS), hydroxymethylglutaryl-CoA reductase (HMGR), mevalonate kinase (MK), diphosphomevalonate decarboxylase (MDD), respectively. The AACT, HMGS, HMGR, MK and MDD were key enzymes for the biosynthesis of isopentenyl diphosphate (IPP) in the mevalonate pathway (Miziorko 2011). Isopentenyl-diphosphate delta-isomerase (IPI), farnesyl diphosphate synthase (FDPS) and geranylgeranyl diphosphate synthase (GGPPs) regulated the biosynthesis flux of geranylgeranyl diphosphate (Geranygeranyl-PP, GGPP), a universal precursor for diterpene biosynthesis. Unigenes of c11713_g1, c10023_g1 and c1886_g1 were homologous to the IPI gene, FDPS gene and GGPPs gene, respectively. Additionally, 40 unigenes were annotated to be involved in the biosynthetic steps from GGPP to taxol within ‘diterpenoid biosynthesis’ pathway.

### Prediction of one fungal taxol biosynthesis pathway

Among the 40 unigenes involved in the taxol biosynthesis of *C. cladosporioides* MD2 (Fig. 5; Additional file 1: Table S4), unigene c11040_g1 was homologous to a gene for taxadiene synthase (TS), that catalyzed the cyclization of GGPP into taxa-4(5),11(12)-diene (Wildung and Croteau 1996), one committed step in the construction of the unique taxane skeleton for the biosynthesis of taxol and related taxoids (Walker and Croteau 2001). Twenty-one unigenes were homologous to a gene for taxadiene 5-alpha-hydroxylase (T5αH), that catalyzed the hydroxylation of taxadiene C5 to taxadienol (Jennewein et al. 2004). Seventeen unigenes were homologous to a gene for taxane 13-alpha-hydroxylase (T13αH), that catalyzed the hydroxylation of taxane C13 (Jennewein et al. 2001). Unigene c5600_g1 was homologous to a gene for 2-alpha-hydroxytaxane 2-*O*-benzoyltransferase (TBT), that catalyzed the conversion of a 2-debenzoyl ‘taxoid-type’ intermediate to 10-deacetylbaccatin III (10-DAB), a late-stage acylation step in the taxol biosynthetic pathway (Walker and Croteau 2000a).

Correspondingly, a potential and partial taxol biosynthetic pathway from GGPP to 10-DAB in the *C. cladosporioides* MD2 transcriptome was speculated (Fig. 5). The GGPP was cyclized by TS into taxa-4(5),11(12)-diene, and further catalyzed by T5αH into taxa-4(20),11(12)-dien-5α-*ol*. Then, the taxa-4(20),11(12)-dien-5α-*ol* was catalyzed by T13αH into taxa-4(20),11(12)-dien-5α,13α-diol, and followed to synthesize 10-deacetyl-2-debenzoylbaccatin III by several undefined steps, which might be a novel and potential biosynthesis branch of 10-deacetyl-2-debenzoylbaccatin III in *C. cladosporioides* MD2. Finally, the 10-deacetyl-2-debenzoylbaccatin III was catalyzed by TBT into 10-DAB. These unigenes homologous to the genes for TS, T5αH, T13αH and TBT provided candidates for further verification of the fungal taxol biosynthesis pathway in *C. cladosporioides* MD2.

## Discussion

A fact was that successive replating of most of taxol-producing fungi in semisynthetic medium resulted in a decrease in taxol production (Flores-Bustamante et al. 2010; Venugopalan and Srivastava 2015). The original taxol yield and 10-DAB yield of *C. cladosporioides* MD2 were about 800 and 120 µg/L in 2009 (Zhang et al. 2009), however they were dramatically decreased to about 5–7 and 50 µg/L in 2014, respectively. Afterwards, the taxol yield and 10-DAB yield of *C. cladosporioides* MD2 basically remained unchanged up to the final test in July, 2016. This biological phenomenon of loss in taxol productivity with subculture was certainly related to the taxol biosynthesis mechanism of *C. cladosporioides* MD2. Here, the transcriptome of the successively subcultured *C. cladosporioides* MD2 with taxol production drawdown was investigated for the first time with a RNA-seq technology to explore gene-expressed characteristics in this situation. About 1.77 Gbp clean reads were generated from the transcriptome of *C. cladosporioides* MD2 and further assembled into 16,603 unigenes, that were annotated with seven protein and nucleic databases. Based on the KEGG analysis, 40 unigenes in the *C. cladosporioides* MD2 transcriptome were annotated to be related to the fungal taxol biosynthesis, and were homologous to the genes for TS, T5αH, T13αH, and TBT, respectively.

According to the proved taxol biosynthesis pathway in *Taxus* (Jennewein and Croteau 2001; Walker and Croteau 2001; Croteau et al. 2006; Guo et al. 2006; Liu et al. 2016), GGPP was cyclized by TS into taxa-4(5), 11(12)-diene, that was catalyzed by T5αH into taxa-4(20), 11(12)-dien-5α-*ol*, where two branches arose (Jennewein and Croteau 2001). One recognized branch was that the taxa-4(20), 11(12)-dien-5α-*ol* was catalyzed by taxa-4(20),11(12)-dien-5α-ol-*O*-acetyltransferase (TAT) into taxa-4(20),11(12)-dien-5α-yl acetate and further catalyzed by taxane 10β-hydroxylase (T10βH) into taxa-4(20),11(12)-dien-5α-acetoxy-10β-*ol*. Subsequently, the taxa-4(20),11(12)-dien-5α-acetoxy-10β-*ol* was catalyzed by several undefined steps into 2-debenzoyltaxane, that was hydrolyzed by TBT to produce 10-DAB. However, none of unigenes homologous to the TAT gene and T10βH gene were detected in the *C. cladosporioides* MD2 transcriptome (Fig. 5), suggesting that *C. cladosporioides* MD2 might not have this same branch as the recognized branch of *Taxus*. Another indistinct branch arised from taxa-4(20), 11(12)-dien-5α-*ol* was that the taxa-4(20), 11(12)-dien-5α-*ol* was catalyzed by T13αH into taxa-4(20),11(12)-dien-5α,13α-diol, that was subsequently catalyzed by several indeterminate enzymatic steps to directly synthesize taxol (Jennewein and Croteau 2001); Or, the taxa-4(20), 11(12)-dien-5α-*ol* was catalyzed by T13αH into taxa-4(20),11(12)-dien-5α,13α-diol and subsequently catalyzed by several indeterminate steps into 2-debenzoyltaxane, that was hydrolyzed by TBT to produce 10-DAB (Liu et al. 2016). A total of 17 unigenes homologous to the T13αH gene and one unigene homologous to the TBT gene were detected in the *C. cladosporioides* MD2 transcriptome, and this branch participated by T13αH and TBT might be a novel and potential branch for the 10-deacetyl-2-debenzoylbaccatin III biosynthesis of *C. cladosporioides* MD2.

Additionally, an interesting and important finding was that unigenes homologous to those genes for enzymes functioning in the biosynthetic steps from 10-DAB to taxol, such as 10-deacetylbaccatin III-10-*O*-acetyltransferase (DBAT), C-13 phenylpropanoid side chain-CoA acyltransferase (BAPT) and 3′-*N*-debenzoyltaxol N-benzoyltransferase (DBTNBT), were not detected in the *C. cladosporioides* MD2 transcriptome. DBAT catalyzed the 10-DAB into Baccatin III, one immediate diterpenoid precursor of taxol (Walker and Croteau 2000b), BAPT catalyzed the selective 13-*O*-acylation of Baccatin III with β-phenylalanoyl-CoA as one acyl donor to form *N*-debenzoyl-2′-deoxytaxol (i.e. catalyzed the attachment of biologically important taxol side chain precursor) (Walker et al. 2002a), and DBTNBT catalyzed the final acylation reaction of taxol biosynthesis (Walker et al. 2002b). None of candidate unigenes homologous to those genes for DBAT, BAPT and DBTNBT might be a reason for the loss of taxol productivity in the subcultured *C. cladosporioides* MD2. This result clued us for future research that, (1) whether some genes homologous to the genes for DBAT, BAPT and DBTNBT exited in the genome but did not transcribed in the transcriptome of the subcultured *C. cladosporioides* MD2; (2) Or, some genes, that encoded products with similar function as DBAT, BAPT, DBTNBT but had low sequence identities to their genes, were not annotated and detected by sequence alignment in the transcriptome of *C. cladosporioides* MD2.

Collectively, this study not only provided an available transcriptome resource for further functional genomic research and comparative transcriptome analysis of *C. cladosporioides* MD2, but also revealed one potential and novel branch for the 10-deacetyl-2-debenzoylbaccatin III biosynthesis and valuable candidate genes for future investigation of the taxol biosynthesis mechanism in *C. cladosporioides* MD2.

## Additional file

**Additional file 1: Figure S1.** Reverse-transcribed PCR validation of the *C. cladosporioides* MD2 unigenes. Ten of the *C. cladosporioides* MD2 unigenes were randomly selected for reverse-transcribed PCR (RT-PCR) validation. The results showed that these selected unigenes got right amplifications. M: DL2000 marker; lane 1-10 was the amplification products of unigenes of c3348_g1, c14778_g1, c2571_g1, c10254_g1, c11322_g1, c6738_g1, c13171_g1, c11554_g1, c13227_g1 and c13492_g1, respectively. **Table S1.** The primers of the ten unigenes for Reverse-Transcribed PCR (RT-PCR) validation. **Table S2.** GO annotation of the *C. cladosporioides* MD2 unigenes. **Table S3.** KEGG classification of the *C. cladosporioides* MD2 unigenes. **Table S4.** Enzymes and related unigenes in the potential fungal taxol biosynthesis pathway of *C. cladosporioides* MD2.

## Abbreviations

10-DAB: 10-deacetylbaccatin III
AACT: acetyl-CoA C-acetyltransferase
BAPT: C-13 phenylpropanoid side chain-CoA acyltransferase
DBAT: 10-deacetylbaccatin III-10-*O*-acetyltransferase
DBTNBT: 3′-*N*-debenzoyltaxol *N*-benzoyltransferase
FDPS: farnesyl diphosphate synthase
GGPP: geranylgeranyl diphosphate
GGPPs: geranylgeranyl diphosphate synthase
HMGR: hydroxymethylglutaryl-CoA reductase
HMGS: hydroxymethylglutaryl-CoA synthase
IPI: isopentenyl-diphosphate delta-isomerase
IPP: isopentenyl diphosphate
MDD: diphosphomevalonate decarboxylase
MK: mevalonate kinase
T10βH: taxane 10β-hydroxylase
T13αH: taxane 13-alpha-hydroxylase
T5αH: taxadiene 5-alpha-hydroxylase
TAT: taxa-4(20),11(12)-dien-5α-*ol*-*O*-acetyltransferase
TBT: 2-alpha-hydroxytaxane 2-*O*-benzoyltransferase
TS: taxadiene synthase
