# Supplementary material for: Transcriptome analysis of a taxol-producing endophytic fungus Cladosporium cladosporioides MD2
Source: AMB Express. 2018 Mar 19;8:41. doi: 10.1186/s13568-018-0567-6 (PMC5859003; doi:10.1186/s13568-018-0567-6)
Supplement: Supplementary file 1 — Additional file 1: Figure S1. Reverse-transcribed PCR validation of the C. cladosporioides MD2 unigenes. Ten of the C. cladosporioides MD2 unigenes were randomly selected for reverse-transcribed PCR (RT-PCR) validation. The results showed that these selected unigenes got right amplifications. M: DL2000 marker; lane 1-10 was the amplification products of unigenes of c3348_g1, c14778_g1, c2571_g1, c10254_g1, c11322_g1, c6738_g1, c13171_g1, c11554_g1, c13227_g1 and c13492_g1, respectively. Table S1. The primers of the ten unigenes for Reverse-Transcribed PCR (RT-PCR) validation. Table S2. GO annotation of the C. cladosporioides MD2 unigenes. Table S3. KEGG classification of the C. cladosporioides MD2 unigenes. Table S4. Enzymes and related unigenes in the potential fungal taxol biosynthesis pathway of C. cladosporioides MD2. [file 13568_2018_567_MOESM1_ESM.pdf]

Applied Microbiology and Biotechnology (transfer to AMB Express)

Transcriptome analysis of a taxol-producing endophytic fungus *Cladosporium cladosporioides* MD2

Li-Yun Miao [1,2,3+], Xin-Chun Mo [4+], Xiao-Yuan Xi [1+], Lan Zhou[1], Ge De[1], You-Sheng Ke[1], Pan Liu[1], Fa-Jun Song[1], Wen-Wen Jin[3*], Peng Zhang[1*]

[1] Hubei Provincial Key Laboratory for Protection and Application of Special Plants in Wuling Area of China, Key Laboratory of State Ethnic Affairs Commission for Biological Technology, College of Life Science, South-Central University for Nationalities, Wuhan 430074, China;

[2] School of Life Science, Wuchang University of Technology, Wuhan 430074, China;

[3] School of Life Science and Technology, Huazhong University of Science and Technology. Wuhan 430074, China;

[4] School of Applied Technology, Lijiang Teacher College. Lijiang, 674100, China

[+] These authors contributed equally to this work.

Corresponding authors:

Peng Zhang

College of Life Science, South-Central University for Nationalities, Wuhan 430074, China;

Email: zhangpenghust@126.com;

Telephone and fax numbers: +86 (27) 67842689.

Wen-Wen Jin

School of Life Science and Technology, Huazhong University of Science and Technology. Wuhan 430074, China;

Email: jww@hust.edu.cn;

Telephone and fax numbers: +86 (27) 87792264.

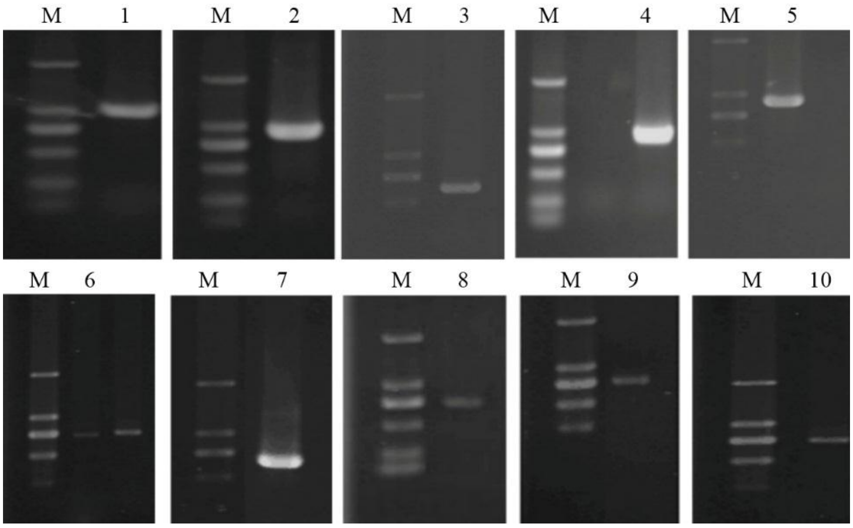

Fig. S1. Reverse-transcribed PCR validation of the *C. cladosporioides* MD2 unigenes. Ten of the *C. cladosporioides* MD2 unigenes were randomly selected for reverse-transcribed PCR (RT-PCR) validation. The results showed that these selected unigenes got right amplifications. M: DL2000 marker; lane 1-10 was the amplification products of unigenes of c3348_g1, c14778_g1, c2571_g1, c10254_g1, c11322_g1, c6738_g1, c13171_g1, c11554_g1, c13227_g1 and c13492_g1, respectively.

Table S1. The primers of the ten unigenes for Reverse-Transcribed PCR (RT-PCR) validation.

Unigene IDs	Primers
c13492_g1	Forward: 5'- ATGCTGCTCGCTTCTACCCTC -3'<br>Reverse: 5'- TCAGTCCAGGGAACCGGGATGCG -3'
c13227_g1	Forward: 5'- ATGGCCTTGGTTTATCGCAA -3'<br>Reverse: 5'- CTACCTCACAATCACCACCG -3'
c11554_g1	Forward: 5'- ATGGAAGGCGTGACATAT -3'<br>Reverse: 5'- TCATGACAGCAGGGAGCC -3'
c13171_g1	Forward: 5'- ATGACTGGCGCGAGAAAAG -3'<br>Reverse: 5'- TCAGTCCGCGAAAGTTCTTG -3'
c3348_g1	Forward: 5'- ATGCTCGCAAGCATTGCTTTTTCTG -3'<br>Reverse: 5'- TTAAGCAGTCGGATACGGGTAGTTC -3'
c14778_g1	Forward: 5'- ATGCCTTATTTCAACATCTGCG -3'<br>Reverse: 5'- TCACCAGAACACCCCATTGATAG -3'
c2571_g1	Forward: 5'- ATGTCGCTGTTTGAGCCC -3'<br>Reverse: 5'- TCACTTACCACCGCCAATG -3'
c10254_g1	Forward: 5'- ATGGCCGGAATGGCAATC -3'<br>Reverse: 5'- CTACAATCTCGCCTTGGG -3'
c11322_g1	Forward: 5'- ATGCACGCTTTCGCAATCT -3'<br>Reverse: 5'- TCAAGCAGAGCATTTCCCTT -3'
c6738_g1	Forward: 5'- ATGAGTCTGCAGGTGCCCGTC -3'<br>Reverse: 5'- TTACATGATGATCTCGTTTTGTACG -3'

Table S2. GO annotation of the *C. cladosporioides* MD2 unigenes.

GO ID (Lev2)	GO Term (Lev2)	GO Term (Lev1)	Gene Number
GO:0005215	transporter activity	Molecular Function	726
GO:0030234	enzyme regulator activity	Molecular Function	105
GO:0098772	molecular function regulator	Molecular Function	170
GO:0016530	metallochaperone activity	Molecular Function	4
GO:0003824	catalytic activity	Molecular Function	4156
GO:0005198	structural molecule activity	Molecular Function	245
GO:0016247	channel regulator activity	Molecular Function	34
GO:0060089	molecular transducer activity	Molecular Function	167
GO:0005488	binding	Molecular Function	4519
GO:0030545	receptor regulator activity	Molecular Function	9
GO:0016209	antioxidant activity	Molecular Function	49
GO:0005085	guanyl-nucleotide exchange factor activity	Molecular Function	12
GO:0000988	protein binding transcription factor activity	Molecular Function	118
GO:0001071	nucleic acid binding transcription factor activity	Molecular Function	415
GO:0032501	multicellular organismal process	Biological Process	132

GO:0009987	cellular process	Biological Process	4944
GO:0040007	growth	Biological Process	5
GO:0048518	positive regulation of biological process	Biological Process	109
GO:0044699	single-organism process	Biological Process	4090
GO:0071840	cellular component organization or biogenesis	Biological Process	795
GO:0007610	behavior	Biological Process	16
GO:0008152	metabolic process	Biological Process	4861
GO:0048519	negative regulation of biological process	Biological Process	78
GO:0001906	cell killing	Biological Process	6
GO:0065007	biological regulation	Biological Process	1627
GO:0098743	cell aggregation	Biological Process	1
GO:0032502	developmental process	Biological Process	96
GO:0050789	regulation of biological process	Biological Process	1496
GO:0022610	biological adhesion	Biological Process	60
GO:0048511	rhythmic process	Biological Process	4
GO:0000003	reproduction	Biological Process	65
GO:0051704	multi-organism process	Biological Process	443
GO:0044848	biological phase	Biological Process	3
GO:0051179	localization	Biological Process	1583
GO:0022414	reproductive process	Biological Process	40
GO:0040011	locomotion	Biological Process	82
GO:0023052	signaling	Biological Process	574
GO:0050896	response to stimulus	Biological Process	1037
GO:0002376	immune system process	Biological Process	41
GO:0031974	membrane-enclosed lumen	Cellular Component	294
GO:0044422	organelle part	Cellular Component	898
GO:0044464	cell part	Cellular Component	2707
GO:0044420	extracellular matrix component	Cellular Component	14
GO:0005576	extracellular region	Cellular Component	146
GO:0045202	synapse	Cellular Component	4
GO:0044456	synapse part	Cellular Component	4
GO:0044425	membrane part	Cellular Component	1429
GO:0030054	cell junction	Cellular Component	2
GO:0043226	organelle	Cellular Component	1870
GO:0009295	nucleoid	Cellular Component	1
GO:0044421	extracellular region part	Cellular Component	144
GO:0019012	virion	Cellular Component	239
GO:0032991	macromolecular complex	Cellular Component	1575
GO:0016020	membrane	Cellular Component	1512
GO:0031012	extracellular matrix	Cellular Component	26
GO:0005623	cell	Cellular Component	2707
GO:0044423	virion part	Cellular Component	239

Table S3. KEGG classification of the *C. cladosporioides* MD2 unigenes.

Pathway Hierarchy1	Pathway Hierarchy2	KEGG Pathway	Pathway ID	Gene Number
Metabolism	Overview	Biosynthesis of amino acids	ko01230	157
Metabolism	Overview	Carbon metabolism	ko01200	150
Genetic Information Processing	Translation	Ribosome	ko03010	104
Genetic Information Processing	Transcription	Spliceosome	ko03040	97
Metabolism	Nucleotide metabolism	Purine metabolism	ko00230	79
Metabolism	Carbohydrate metabolism	Starch and sucrose metabolism	ko00500	78
Cellular Processes	Cell growth and death	Cell cycle - yeast	ko04111	78
Genetic Information Processing	Folding, sorting and degradation	Protein processing in endoplasmic reticulum	ko04141	74
Cellular Processes	Transport and catabolism	Peroxisome	ko04146	67
Metabolism	Carbohydrate metabolism	Glycolysis / Gluconeogenesis	ko00010	61
Metabolism	Energy metabolism	Oxidative phosphorylation	ko00190	59
Metabolism	Amino acid metabolism	Glycine, serine and threonine metabolism	ko00260	59
Genetic Information Processing	Translation	RNA transport	ko03013	59
Cellular Processes	Cell growth and death	Meiosis - yeast	ko04113	59
Metabolism	Nucleotide metabolism	Pyrimidine metabolism	ko00240	57
Metabolism	Carbohydrate metabolism	Amino sugar and nucleotide sugar metabolism	ko00520	56
Cellular Processes	Transport and catabolism	Endocytosis	ko04144	55
Genetic Information Processing	Folding, sorting and degradation	Ubiquitin mediated proteolysis	ko04120	54
Cellular Processes	Cell growth and death	Cell cycle	ko04110	53
Metabolism	Carbohydrate metabolism	Glyoxylate and dicarboxylate metabolism	ko00630	52
Metabolism	Overview	2-Oxocarboxylic acid metabolism	ko01210	52
Genetic Information Processing	Translation	Ribosome biogenesis in eukaryotes	ko03008	51
Metabolism	Amino acid metabolism	Arginine and proline metabolism	ko00330	49
Genetic Information Processing	Folding, sorting and degradation	RNA degradation	ko03018	47
Metabolism	Energy metabolism	Methane metabolism	ko00680	46
Metabolism	Overview	Fatty acid metabolism	ko01212	46
Metabolism	Amino acid metabolism	Cysteine and methionine metabolism	ko00270	44
Metabolism	Amino acid metabolism	Phenylalanine metabolism	ko00360	44

Genetic Information Processing	Translation	Aminoacyl-tRNA biosynthesis	ko00970	44
Metabolism	Carbohydrate metabolism	Pyruvate metabolism	ko00620	43
Organismal Systems	Endocrine system	Insulin signaling pathway	ko04910	43
Cellular Processes	Transport and catabolism	Phagosome	ko04145	42
Metabolism	Carbohydrate metabolism	Galactose metabolism	ko00052	40
Metabolism	Amino acid metabolism	Alanine, aspartate and glutamate metabolism	ko00250	40
Environmental Information Processing	Signal transduction	AMPK signaling pathway	ko04152	40
Metabolism	Amino acid metabolism	Valine, leucine and isoleucine degradation	ko00280	39
Metabolism	Carbohydrate metabolism	Fructose and mannose metabolism	ko00051	38
Metabolism	Amino acid metabolism	Tryptophan metabolism	ko00380	38
Metabolism	Biosynthesis of other secondary metabolites	Phenylpropanoid biosynthesis	ko00940	38
Genetic Information Processing	Replication and repair	Nucleotide excision repair	ko03420	38
Cellular Processes	Cell growth and death	Oocyte meiosis	ko04114	38
Metabolism	Lipid metabolism	Glycerophospholipid metabolism	ko00564	37
Genetic Information Processing	Translation	mRNA surveillance pathway	ko03015	37
Environmental Information Processing	Signal transduction	PI3K-Akt signaling pathway	ko04151	37
Metabolism	Lipid metabolism	Glycerolipid metabolism	ko00561	35
Genetic Information Processing	Replication and repair	DNA replication	ko03030	35
Metabolism	Carbohydrate metabolism	Pentose and glucuronate interconversions	ko00040	34
Metabolism	Amino acid metabolism	Tyrosine metabolism	ko00350	34
Metabolism	Glycan biosynthesis and metabolism	N-Glycan biosynthesis	ko00510	34
Metabolism	Carbohydrate metabolism	Butanoate metabolism	ko00650	34
Metabolism	Energy metabolism	Carbon fixation in photosynthetic organisms	ko00710	34
Metabolism	Carbohydrate metabolism	Citrate cycle (TCA cycle)	ko00020	33
Metabolism	Xenobiotics biodegradation and metabolism	Metabolism of xenobiotics by cytochrome P450	ko00980	33
Cellular Processes	Transport and catabolism	Lysosome	ko04142	33
Organismal Systems	Endocrine system	Oxytocin signaling pathway	ko04921	33
Metabolism	Carbohydrate metabolism	Pentose phosphate pathway	ko00030	32

Metabolism	Metabolism of other amino acids	Glutathione metabolism	ko00480	32
Environmental Information Processing	Membrane transport	ABC transporters	ko02010	32
Genetic Information Processing	Folding, sorting and degradation	Proteasome	ko03050	32
Metabolism	Lipid metabolism	Fatty acid degradation	ko00071	30
Metabolism	Metabolism of other amino acids	beta-Alanine metabolism	ko00410	30
Cellular Processes	Cell motility	Regulation of actin cytoskeleton	ko04810	30
Metabolism	Carbohydrate metabolism	Inositol phosphate metabolism	ko00562	29
Environmental Information Processing	Signal transduction	cAMP signaling pathway	ko04024	29
Environmental Information Processing	Signal transduction	HIF-1 signaling pathway	ko04066	29
Genetic Information Processing	Replication and repair	Mismatch repair	ko03430	28
Environmental Information Processing	Signal transduction	Ras signaling pathway	ko04014	28
Organismal Systems	Endocrine system	Thyroid hormone signaling pathway	ko04919	28
Metabolism	Carbohydrate metabolism	Propanoate metabolism	ko00640	27
Environmental Information Processing	Signal transduction	FoxO signaling pathway	ko04068	27
Metabolism	Amino acid metabolism	Phenylalanine, tyrosine and tryptophan biosynthesis	ko00400	26
Metabolism	Lipid metabolism	Biosynthesis of unsaturated fatty acids	ko01040	26
Environmental Information Processing	Signal transduction	MAPK signaling pathway - yeast	ko04011	25
Environmental Information Processing	Signal transduction	Rap1 signaling pathway	ko04015	25
Environmental Information Processing	Signal transduction	Two-component system	ko02020	24
Organismal Systems	Immune system	Chemokine signaling pathway	ko04062	24
Environmental Information Processing	Signal transduction	mTOR signaling pathway	ko04150	24
Cellular Processes	Cellular commiunity	Tight junction	ko04530	24
Organismal Systems	Nervous system	Dopaminergic synapse	ko04728	24
Metabolism	Amino acid metabolism	Lysine degradation	ko00310	23
Organismal Systems	Endocrine system	PPAR signaling pathway	ko03320	23
Environmental Information Processing	Signal transduction	MAPK signaling pathway	ko04010	23

Environmental Information Processing	Signal transduction	Phosphatidylinositol signaling system	ko04070	23
Metabolism	Metabolism of other amino acids	Cyanoamino acid metabolism	ko00460	22
Genetic Information Processing	Replication and repair	Fanconi anemia pathway	ko03460	22
Environmental Information Processing	Signal transduction	cGMP - PKG signaling pathway	ko04022	22
Cellular Processes	Cellular commiunity	Focal adhesion	ko04510	22
Organismal Systems	Endocrine system	Progesterone-mediated oocyte maturation	ko04914	22
Organismal Systems	Digestive system	Bile secretion	ko04976	22
Metabolism	Lipid metabolism	Fatty acid biosynthesis	ko00061	21
Metabolism	Lipid metabolism	Steroid biosynthesis	ko00100	21
Metabolism	Glycan biosynthesis and metabolism	Various types of N-glycan biosynthesis	ko00513	21
Metabolism	Xenobiotics biodegradation and metabolism	Chloroalkane and chloroalkene degradation	ko00625	21
Metabolism	Metabolism of cofactors and vitamins	Pantothenate and CoA biosynthesis	ko00770	21
Genetic Information Processing	Transcription	Basal transcription factors	ko03022	21
Environmental Information Processing	Signal transduction	Wnt signaling pathway	ko04310	21
Organismal Systems	Nervous system	Synaptic vesicle cycle	ko04721	21
Organismal Systems	Nervous system	Glutamatergic synapse	ko04724	21
Organismal Systems	Excretory system	Vasopressin-regulated water reabsorption	ko04962	21
Genetic Information Processing	Replication and repair	Base excision repair	ko03410	20
Organismal Systems	Circulatory system	Adrenergic signaling in cardiomyocytes	ko04261	20
Organismal Systems	Immune system	Fc gamma R-mediated phagocytosis	ko04666	20
Metabolism	Amino acid metabolism	Valine, leucine and isoleucine biosynthesis	ko00290	19
Metabolism	Metabolism of cofactors and vitamins	Porphyrin and chlorophyll metabolism	ko00860	19
Metabolism	Energy metabolism	Sulfur metabolism	ko00920	19
Metabolism	Xenobiotics biodegradation and metabolism	Drug metabolism - cytochrome P450	ko00982	19

Organismal Systems	Nervous system	Neurotrophin signaling pathway	ko04722	19
Organismal Systems	Nervous system	GABAergic synapse	ko04727	19
Organismal Systems	Endocrine system	GnRH signaling pathway	ko04912	19
Organismal Systems	Endocrine system	Estrogen signaling pathway	ko04915	19
Metabolism	Glycan biosynthesis and metabolism	Glycosylphosphatidylinositol(GPI)-anchor biosynthesis	ko00563	18
Metabolism	Overview	Degradation of aromatic compounds	ko01220	18
Organismal Systems	Immune system	T cell receptor signaling pathway	ko04660	18
Organismal Systems	Immune system	Leukocyte transendothelial migration	ko04670	18
Organismal Systems	Nervous system	Long-term potentiation	ko04720	18
Metabolism	Energy metabolism	Photosynthesis	ko00195	17
Metabolism	Amino acid metabolism	Lysine biosynthesis	ko00300	17
Metabolism	Lipid metabolism	Arachidonic acid metabolism	ko00590	17
Metabolism	Metabolism of cofactors and vitamins	Nicotinate and nicotinamide metabolism	ko00760	17
Metabolism	Metabolism of cofactors and vitamins	Retinol metabolism	ko00830	17
Metabolism	Biosynthesis of other secondary metabolites	Tropane, piperidine and pyridine alkaloid biosynthesis	ko00960	17
Genetic Information Processing	Transcription	RNA polymerase	ko03020	17
Organismal Systems	Development	Axon guidance	ko04360	17
Environmental Information Processing	Signal transduction	Hippo signaling pathway	ko04390	17
Cellular Processes	Cellular commiunity	Gap junction	ko04540	17
Organismal Systems	Nervous system	Serotonergic synapse	ko04726	17
Genetic Information Processing	Replication and repair	Homologous recombination	ko03440	16
Environmental Information Processing	Signal transduction	ErbB signaling pathway	ko04012	16
Organismal Systems	Immune system	Platelet activation	ko04611	16
Organismal Systems	Nervous system	Retrograde endocannabinoid signaling	ko04723	16
Organismal Systems	Endocrine system	Melanogenesis	ko04916	16
Metabolism	Carbohydrate metabolism	Ascorbate and aldarate metabolism	ko00053	15
Metabolism	Metabolism of cofactors and vitamins	One carbon pool by folate	ko00670	15
Metabolism	Metabolism of cofactors and vitamins	Biotin metabolism	ko00780	15

Metabolism	Metabolism of cofactors and vitamins	Folate biosynthesis	ko00790	15
Metabolism	Biosynthesis of other secondary metabolites	Isoquinoline alkaloid biosynthesis	ko00950	15
Organismal Systems	Nervous system	Cholinergic synapse	ko04725	15
Organismal Systems	Endocrine system	Adipocytokine signaling pathway	ko04920	15
Metabolism	Metabolism of cofactors and vitamins	Ubiquinone and other terpenoid-quinone biosynthesis	ko00130	14
Metabolism	Lipid metabolism	alpha-Linolenic acid metabolism	ko00592	14
Metabolism	Lipid metabolism	Sphingolipid metabolism	ko00600	14
Metabolism	Energy metabolism	Carbon fixation pathways in prokaryotes	ko00720	14
Metabolism	Metabolism of terpenoids and polyketides	Terpenoid backbone biosynthesis	ko00900	14
Metabolism	Energy metabolism	Nitrogen metabolism	ko00910	14
Environmental Information Processing	Signal transduction	Calcium signaling pathway	ko04020	14
Organismal Systems	Circulatory system	Vascular smooth muscle contraction	ko04270	14
Environmental Information Processing	Signal transduction	VEGF signaling pathway	ko04370	14
Cellular Processes	Cellular commiunity	Adherens junction	ko04520	14
Organismal Systems	Excretory system	Endocrine and other factor-regulated calcium reabsorption	ko04961	14
Metabolism	Amino acid metabolism	Histidine metabolism	ko00340	13
Metabolism	Lipid metabolism	Ether lipid metabolism	ko00565	13
Metabolism	Xenobiotics biodegradation and metabolism	Aminobenzoate degradation	ko00627	13
Cellular Processes	Cell growth and death	p53 signaling pathway	ko04115	13
Cellular Processes	Transport and catabolism	Regulation of autophagy	ko04140	13
Organismal Systems	Immune system	Antigen processing and presentation	ko04612	13
Organismal Systems	Immune system	Natural killer cell mediated cytotoxicity	ko04650	13
Organismal Systems	Nervous system	Long-term depression	ko04730	13
Organismal Systems	Endocrine system	Thyroid hormone synthesis	ko04918	13
Organismal Systems	Digestive system	Pancreatic secretion	ko04972	13
Metabolism	Glycan biosynthesis and metabolism	Other glycan degradation	ko00511	12

Metabolism	Xenobiotics biodegradation and metabolism	Styrene degradation	ko00643	12
Organismal Systems	Circulatory system	Cardiac muscle contraction	ko04260	12
Organismal Systems	Environmental adaptation	Plant-pathogen interaction	ko04626	12
Organismal Systems	Environmental adaptation	Circadian entrainment	ko04713	12
Organismal Systems	Sensory system	Inflammatory mediator regulation of TRP channels	ko04750	11
Organismal Systems	Excretory system	Collecting duct acid secretion	ko04966	11
Organismal Systems	Digestive system	Gastric acid secretion	ko04971	11
Metabolism	Energy metabolism	Photosynthesis - antenna proteins	ko00196	10
Metabolism	Xenobiotics biodegradation and metabolism	Drug metabolism - other enzymes	ko00983	10
Genetic Information Processing	Folding, sorting and degradation	Protein export	ko03060	10
Environmental Information Processing	Signal transduction	TGF-beta signaling pathway	ko04350	10
Environmental Information Processing	Signal transduction	Hippo signaling pathway -fly	ko04391	10
Organismal Systems	Immune system	B cell receptor signaling pathway	ko04662	10
Organismal Systems	Environmental adaptation	Circadian rhythm	ko04710	10
Metabolism	Lipid metabolism	Fatty acid elongation	ko00062	9
Metabolism	Xenobiotics biodegradation and metabolism	Benzoate degradation	ko00362	9
Metabolism	Metabolism of other amino acids	Taurine and hypotaurine metabolism	ko00430	9
Metabolism	Metabolism of other amino acids	Selenocompound metabolism	ko00450	9
Metabolism	Lipid metabolism	Linoleic acid metabolism	ko00591	9
Metabolism	Xenobiotics biodegradation and metabolism	Naphthalene degradation	ko00626	9
Genetic Information Processing	Folding, sorting and degradation	Sulfur relay system	ko04122	9
Organismal Systems	Immune system	Cytosolic DNA-sensing pathway	ko04623	9
Metabolism	Metabolism of other amino acids	Phosphonate and phosphinate metabolism	ko00440	8
Metabolism	Biosynthesis of other secondary metabolites	Streptomycin biosynthesis	ko00521	8

Metabolism	Metabolism of cofactors and vitamins	Riboflavin metabolism	ko00740	8
Metabolism	Xenobiotics biodegradation and metabolism	Caprolactam degradation	ko00930	8
Cellular Processes	Cell growth and death	Apoptosis	ko04210	8
Environmental Information Processing	Signal transduction	Hedgehog signaling pathway	ko04340	8
Organismal Systems	Immune system	Fc epsilon RI signaling pathway	ko04664	8
Organismal Systems	Endocrine system	Insulin secretion	ko04911	8
Organismal Systems	Endocrine system	Prolactin signaling pathway	ko04917	8
Organismal Systems	Digestive system	Salivary secretion	ko04970	8
Metabolism	Xenobiotics biodegradation and metabolism	Bisphenol degradation	ko00363	7
Metabolism	Metabolism of cofactors and vitamins	Thiamine metabolism	ko00730	7
Genetic Information Processing	Folding, sorting and degradation	SNARE interactions in vesicular transport	ko04130	7
Organismal Systems	Development	Osteoclast differentiation	ko04380	7
Organismal Systems	Immune system	RIG-I-like receptor signaling pathway	ko04622	7
Organismal Systems	Sensory system	Phototransduction - fly	ko04745	7
Organismal Systems	Excretory system	Aldosterone-regulated sodium reabsorption	ko04960	7
Organismal Systems	Digestive system	Carbohydrate digestion and absorption	ko04973	7
Metabolism	Carbohydrate metabolism	C5-Branched dibasic acid metabolism	ko00660	6
Metabolism	Metabolism of cofactors and vitamins	Vitamin B6 metabolism	ko00750	6
Environmental Information Processing	Signal transduction	MAPK signaling pathway - fly	ko04013	6
Organismal Systems	Endocrine system	Renin-angiotensin system	ko04614	6
Metabolism	Lipid metabolism	Synthesis and degradation of ketone bodies	ko00072	5
Metabolism	Lipid metabolism	Primary bile acid biosynthesis	ko00120	5
Metabolism	Lipid metabolism	Steroid hormone biosynthesis	ko00140	5
Metabolism	Glycan biosynthesis and metabolism	Other types of O-glycan biosynthesis	ko00514	5
Environmental Information Processing	Signal transduction	NF-kappa B signaling pathway	ko04064	5
Organismal Systems	Development	Dorso-ventral axis formation	ko04320	5

Organismal Systems	Immune system	NOD-like receptor signaling pathway	ko04621	5
Environmental Information Processing	Signal transduction	TNF signaling pathway	ko04668	5
Organismal Systems	Sensory system	Olfactory transduction	ko04740	5
Organismal Systems	Excretory system	Proximal tubule bicarbonate reclamation	ko04964	5
Organismal Systems	Digestive system	Protein digestion and absorption	ko04974	5
Organismal Systems	Digestive system	Fat digestion and absorption	ko04975	5
Metabolism	Biosynthesis of other secondary metabolites	Butirosin and neomycin biosynthesis	ko00524	4
Metabolism	Metabolism of terpenoids and polyketides	Limonene and pinene degradation	ko00903	4
Genetic Information Processing	Replication and repair	Non-homologous end-joining	ko03450	4
Environmental Information Processing	Signal transduction	Notch signaling pathway	ko04330	4
Organismal Systems	Immune system	Toll-like receptor signaling pathway	ko04620	4
Metabolism	Biosynthesis of other secondary metabolites	Novobiocin biosynthesis	ko00401	3
Metabolism	Glycan biosynthesis and metabolism	Glycosaminoglycan degradation	ko00531	3
Metabolism	Glycan biosynthesis and metabolism	Glycosphingolipid biosynthesis - ganglio series	ko00604	3
Metabolism	Metabolism of terpenoids and polyketides	Biosynthesis of ansamycins	ko01051	3
Environmental Information Processing	Signal transduction	Plant hormone signal transduction	ko04075	3
Cellular Processes	Cell growth and death	Cell cycle - Caulobacter	ko04112	3
Organismal Systems	Environmental adaptation	Circadian rhythm - plant	ko04712	3
Organismal Systems	Sensory system	Taste transduction	ko04742	3
Organismal Systems	Sensory system	Phototransduction	ko04744	3
Organismal Systems	Endocrine system	Ovarian Steroidogenesis	ko04913	3
Organismal Systems	Digestive system	Vitamin digestion and absorption	ko04977	3
Metabolism	Biosynthesis of other secondary metabolites	Caffeine metabolism	ko00232	2
Metabolism	Metabolism of other amino acids	D-Glutamine and D-glutamate metabolism	ko00471	2
Metabolism	Glycan biosynthesis and metabolism	Peptidoglycan biosynthesis	ko00550	2

Metabolism	Glycan biosynthesis and metabolism	Glycosphingolipid biosynthesis - globo series	ko00603	2
Metabolism	Metabolism of cofactors and vitamins	Lipoic acid metabolism	ko00785	2
Metabolism	Metabolism of terpenoids and polyketides	Sesquiterpenoid and triterpenoid biosynthesis	ko00909	2
Metabolism	Biosynthesis of other secondary metabolites	Flavonoid biosynthesis	ko00941	2
Metabolism	Biosynthesis of other secondary metabolites	Stilbenoid, diarylheptanoid and gingerol biosynthesis	ko00945	2
Metabolism	Biosynthesis of other secondary metabolites	Betalain biosynthesis	ko00965	2
Metabolism	Metabolism of terpenoids and polyketides	Nonribosomal peptide structures	ko01054	2
Organismal Systems	Digestive system	Mineral absorption	ko04978	2
Metabolism	Biosynthesis of other secondary metabolites	Aflatoxin biosynthesis	ko00254	1
Metabolism	Metabolism of terpenoids and polyketides	Geraniol degradation	ko00281	1
Metabolism	Xenobiotics biodegradation and metabolism	Chlorocyclohexane and chlorobenzene degradation	ko00361	1
Metabolism	Xenobiotics biodegradation and metabolism	Fluorobenzoate degradation	ko00364	1
Metabolism	Metabolism of terpenoids and polyketides	Polyketide sugar unit biosynthesis	ko00523	1
Metabolism	Xenobiotics biodegradation and metabolism	Toluene degradation	ko00623	1
Metabolism	Biosynthesis of other secondary metabolites	Indole alkaloid biosynthesis	ko00901	1
Metabolism	Metabolism of terpenoids and polyketides	Brassinosteroid biosynthesis	ko00905	1
Metabolism	Metabolism of terpenoids and polyketides	Carotenoid biosynthesis	ko00906	1
Metabolism	Metabolism of terpenoids and polyketides	Zeatin biosynthesis	ko00908	1
Metabolism	Biosynthesis of other secondary metabolites	Isoflavonoid biosynthesis	ko00943	1
Metabolism	Biosynthesis of other secondary metabolites	Flavone and flavonol biosynthesis	ko00944	1
Metabolism	Biosynthesis of other secondary metabolites	Glucosinolate biosynthesis	ko00966	1

Metabolism	Metabolism of terpenoids and polyketides	Insect hormone biosynthesis	ko00981	1
Metabolism	Metabolism of terpenoids and polyketides	Biosynthesis of vancomycin group antibiotics	ko01055	1
Environmental Information Processing	Membrane transport	Bacterial secretion system	ko03070	1
Environmental Information Processing	Signaling molecules and interaction	Neuroactive ligand-receptor interaction	ko04080	1
Environmental Information Processing	Signal transduction	Jak-STAT signaling pathway	ko04630	1
Organismal Systems	Environmental adaptation	Circadian rhythm - fly	ko04711	1

Table S4. Enzymes and related unigenes in the potential fungal taxol biosynthesis pathway of *C. cladosporioides* MD2.

Abbreviation of enzymes	Enzymes		Number	Unigene IDs
AACT	acetyl-CoA C-acetyltransferase	EC:2.3.1.9	2	c12899_g1,c8934_g1
HMGS	hydroxymethylglutaryl-CoA synthase	EC:2.3.3.10	1	c13511_g1
HMGR	hydroxymethylglutaryl-CoA reductase (NADPH)	EC:1.1.1.34	1	c14615_g1
MK	mevalonate kinase	EC:2.7.1.36	1	c12988_g1
MDD	diphosphomevalonate decarboxylase	EC:4.1.1.33	1	c10355_g1
IPI	isopentenyl-diphosphate Delta-isomerase	EC:5.3.3.2	1	c11713_g1
FDPS	farnesyl diphosphate synthase	EC:2.5.1.1	1	c10023_g1
GGPPs	geranylgeranyl diphosphate synthase	EC:2.5.1.29	1	c1886_g1
TS	taxadiene synthase	EC:4.2.3.17	1	c11040_g1
T5αH	taxadiene 5-alpha-hydroxylase	EC:1.14.99.37	21	c9962_g1, c12628_g1, c2186_g1, c8872_g1, c6331_g1, c2364_g1, c11767_g1, c6828_g1, c5652_g1, c6036_g1, c15016_g1, c1021_g1, c13571_g1, c1481_g1, c4632_g3 , c4707_g1, c5994_g1, c5767_g1, c5237_g1, c3501_g1, c240_g1
T13αH	taxane 13-alpha-hydroxylase	EC:1.14.13.77	17	c2078_g1, c2045_g1, c11556_g1, c15030_g1, c4799_g1, c4952_g1,

				c3717_g1, c14211_g1 , c22_g1, c10076_g1, c14298_g1, c3976_g1, c13288_g1, c11506_g1, c6963_g1, c8724_g1, c5859_g1
TBT	2-alpha-hydroxytaxane 2-O-benzoyltransferase	EC:2.3.1.166	1	c5600_g1
